# A Phase I Randomized Placebo Controlled Trial of the Safety of 3% SPL7013 Gel (VivaGel®) in Healthy Young Women Administered Twice Daily for 14 Days

**DOI:** 10.1371/journal.pone.0016258

**Published:** 2011-01-20

**Authors:** Craig R. Cohen, Joelle Brown, Anna-Barbara Moscicki, Elizabeth A. Bukusi, Jeremy R. A. Paull, Clare F. Price, Stephen Shiboski

**Affiliations:** 1 Department of Obstetrics, Gynecology and Reproductive Science, University of California San Francisco, San Francisco, California, United States of America; 2 Department of Epidemiology, University of California Los Angeles, Los Angeles, California, United States of America; 3 Department of Pediatrics, University of California San Francisco, San Francisco, California, United States of America; 4 Center for Microbiology Research, Kenya Medical Research Institute, Nairobi, Kenya; 5 Starpharma Pty Ltd, Melbourne, Australia; 6 Department of Biostatistics, University of California San Francisco, San Francisco, California, United States of America; Tulane University, United States of America

## Abstract

**Objective:**

To assess the safety of VivaGel® used vaginally twice daily for 14 days among healthy, sexually-abstinent women, aged 18–24 years in the USA and Kenya.

**Design:**

Randomized placebo controlled trial.

**Methods:**

Participants were randomized 2∶1, VivaGel to placebo. Safety was assessed by comparing genitourinary (GU) adverse events (AEs), colposcopy findings, vaginal lactobacilli and laboratory abnormalities by arm.

**Results:**

Fifty-four women were enrolled; 35 in the VivaGel arm and 19 in the placebo arm. Twenty-six (74%) and 10 (53%) women reported taking all doses of VivaGel and placebo, respectively. No grade 3 or 4 AEs, or serious AEs occurred. Twenty-five (71%) participants in the VivaGel arm compared to 10 (53%) participants in the placebo arm had at least one grade 1 or 2 GU AE associated with product use (RR = 1.4, 95% CI 0.8-2.2). All seven grade 2 GU AEs associated with product use occurred among four women in the VivaGel arm. Vulvar and cervical erythema, cervical lesions, symptomatic BV, urinary frequency and metrorrhagia were more common in the VivaGel arm than the placebo arm. Twenty-nine (83%) participants in the VivaGel arm had a colposcopic finding compared to 10 (53%) participants in the placebo arm (RR = 1.6, 95%CI = 1.0-2.5). Two women in the VivaGel arm prematurely discontinued product use themselves due to a reported GU AE. Persistence of H_2_O_2_-producing and non-producing lactobacilli did not differ by study arm.

**Conclusions:**

GU AEs and colposcopic findings consistent with mild epithelial irritation and inflammation occurred more commonly among women in the VivaGel arm.

**Trial Registration:**

ClinicalTrials.gov NCT003311032

## Introduction

In sub-Saharan Africa, 59% of people living with HIV are women [1], and in Kenya girls age 15–24 are six times more likely to be HIV-infected than their male age-mates (3% prevalence for girls versus <0.5% for boys) [2]. Furthermore, young women are at a particularly high risk of acquiring genital herpes caused by herpes simplex virus type 2 (HSV-2) and other sexually transmitted infections (STI) which promote the transmission of HIV [3]. A strategy that encourages delayed sexual debut, monogamy and condom use is important to control the spread of STI/HIV, but this approach requires a level of female empowerment and control in sexual relationships often lacking for young women. This widespread disparity of gender power relationships makes the development, evaluation and testing of safe and effective topical microbicides that are effective against HIV and other STIs an urgent priority for young female populations.

VivaGel® (3% w/w SPL7013 in Carbopol®-based aqueous gel) is a microbicide under development by Starpharma Pty Ltd. The active ingredient of VivaGel® is SPL7013 which belongs to the class of macromolecules called dendrimers, characterized by a highly branched three-dimensional architecture [4]. In vitro and in vivo studies of this compound have shown that SPL7013 is a potent inhibitor of HSV-2 and HIV [5]–[8]. SPL7013 is believed to prevent the attachment of HIV to human T-cells by binding glycoprotein-120 and analogously prevent the attachment of HSV-2 to epithelial cells by binding glycoprotein-B. Toxicity studies with 3% SPL 7013 in tissue explants, *in vitro*, animal and non-human primate models have demonstrated relative safety of this product [9]. Three percent SPL 7013, the concentration used in this clinical trial, applied vaginally to pigtailed macaques for four consecutive days did not lead to cervicovaginal tissue disruption and/or friability in any of six animals, while 5% SPL 7013 led to tissue disruption and/or friability in four of six animals [9]. A previous phase 1 trial included colposcopic evaluation in 36 healthy, sexually abstinent,18–45 year old women in Australia and demonstrated safety and tolerance of 0.5% to 3% w/w SPL7013 gel when administered once daily for seven consecutive days [10].

The International Working Group for Microbicides recommends that candidate vaginal microbicides be evaluated in populations with different characteristics [11]. This expanded phase 1 study serves that purpose, in that it complements the other clinical studies conducted to date. The primary objective of this study was to assess the safety and tolerability of VivaGel® (3% w/w SPL7013 Gel) versus placebo (same gel without SPL7013) when applied vaginally two times daily for 14 consecutive days among healthy, HIV and STI free, sexually abstinent, 18–24 year old women in the U.S. and in Kenya.

## Methods

### Ethics Statement

The study protocol and informed consent forms were approved by the Committee on Human Research at UCSF and the National Ethical Review Committee at KEMRI. Safety oversight was provided by Independent Safety Monitors and a Safety Monitoring Committee. 

### Objectives

Safety was assessed by comparing the incidence of adverse events (AE), including genitourinary (GU) clinical signs and symptoms, colposcopic findings of the genital tract, renal and liver function, systemic absorption of SPL7013, and vaginal microflora among participants randomized to VivaGel® (3% w/w SPL7013 in a Carbopol-based aqueous formulation) and those randomized to placebo (Carbopol-based aqueous formulation with 0% w/w SPL7013). The Female Genital Grading Table for Use in Microbicide Studies (November 2007, Addendum 1 to the Division of AIDS (DAIDS) Table for Grading the Severity of Adult Adverse Experiences) was used to grade GU abnormalities, and the definition of GU AEs used in the study were any AEs identified in this table. All AEs had their relationship to product use assessed as either associated (event is temporally related to the administration of product use and no other etiology explains the event) or not associated (the event is temporally independent of the study product and/or the event appears to be explained by another etiology). The WHO/CONRAD colposcopy manual [12] was used to define colposcopic findings, and highly trained staff at both sites performed the speculum examinations. The Division of Microbiology and Infectious Diseases Adult Toxicity Table (December 2004) was used to grade serum chemistry, hematology, and other abnormalities. Clinical signs and symptoms were assessed at enrollment, and 2, 7, 14 and 21 days following initiation of product use; vaginal microflora was assessed at enrollment and 7, 14, and 21 days following initiation of product use, and serum chemistry and hematology were assessed at enrollment and 14 and 21 days following initiation of product use. Participants were discontinued from study product by study staff if a grade 3 or higher AE was reported or discovered during clinical examination during follow-up, or if the participant was non-adherent to the study protocol. Tolerability was assessed by comparing the number of participants in the VivaGel® and placebo arms who discontinued product use themselves due to an overt adverse event. Adherent participants were defined as those who administered the study product twice daily over 14 consecutive days for a total of 28 doses, or those who missed one or two doses on one or two days, but administered these missed doses over one to two additional days to complete the 28 doses.

### Design

This was a Phase 1, placebo-controlled, randomized, double blind, study in sexually-abstinent young women conducted at the Pediatric Clinical Research Center at the University of California, San Francisco (UCSF), USA and the Research Care and Training Program unit of the Center for Microbiology Research at the Kenya Medical Research Institute (KEMRI) in Kisumu, Kenya. Procedures were reviewed by investigators and study personnel at both sites prior to and during study implementation.

### Selection of subjects

Volunteers were enrolled if they were 18-24 years of age and in good health, had regular menstrual cycles of at least 25 days in length, sexually experienced but willing to be sexually abstinent one week prior to enrollment and throughout the 21 days of study participation, not breastfeeding, not pregnant and not within 3 months of last pregnancy outcome, no history of intermenstrual bleeding during the prior three months, and provided written informed consent for study participation. Volunteers who met any of the following criteria were not enrolled: clinically detectable genital epithelia disruption; positive test for human chorionic gonadotropin (hCG), urinary tract infection (UTI), HIV antibodies, HSV-2 antibodies, syphilis, vaginal candidiasis, symptomatic bacterial vaginosis (BV), vaginal Nugent score ≥7 at screening visit, cervicovaginal *Trichomonas vaginalis*, *Neisseria gonorrhoeae, or Chlamydia Trachomatis* at screening or enrollment visits; abnormal cervical cytology (Pap smear); and greater than grade 1 serum chemistry or hematology findings. In addition, exclusion criteria also included allergy to any known component of study product or latex; starting a new long-acting contraceptive treatment (e.g. depomedroxyprogesterone) within the past 3 months; history of recurrent vaginal infections (>2 in past 12 months); active uncontrolled medical condition; acute clinically significant illness within the past 30 days; received a new systemic or topical medication within 14 days prior to using the study product; or currently using any other investigational drug.

### Study procedures

Each volunteer was seen in six scheduled visits. At the first visit (screening), written informed consent was obtained as well as a medical history. A urine sample was obtained to test for hCG and for urinalysis if the volunteer was symptomatic for a UTI. HIV and STI counseling was performed, and a blood sample was collected to test for HIV, HSV-2, syphilis, as well as hematology testing, and liver and renal function testing. A physical examination including pelvic examination was performed. The pelvic examination included naked eye examination and colposcopy, as well as vaginal pH testing, vaginal wet mount for candidiasis, bacterial vaginosis (BV) by Amsel's criteria, and collection of vaginal swabs for lactobacillus culture and Nugent's Gram stain testing for BV [13] and cervicovaginal swabs specimens for *T. vaginalis*, *N. gonorrhoeae, C. trachomatis* testing, and a Pap smear. The volunteer was instructed not to use vaginal products and to abstain from sexual intercourse during the 7 days prior to the second visit.

The second visit (enrollment (Day 0)) was scheduled to fall within 5-14 days after the first day of the next menses. Participants were provided with post-test counseling and screening test results. Eligibility criteria were reviewed, a urine specimen was taken for pregnancy testing and, if the volunteer was symptomatic, for urinalysis. A review of symptoms was conducted, and a pelvic exam including naked eye examination, colposcopy, vaginal pH and vaginal wet mount were performed. If the volunteer continued to meet the eligibility criteria, she was enrolled and additional vaginal specimens were collected for lactobacillus culture, Gram stain and prostate specific antigen (PSA) testing [14], and cervicovaginal swabs specimens for *T. vaginalis*, *N. gonorrhoeae, and C. trachomatis* testing. A blood sample was collected for hematology, liver and renal function testing, and to test for SPL7013 drug levels.

The randomization scheme was developed by a UCSF statistician not otherwise directly involved with study participants. Subjects were randomized in a 2∶1 VivaGel® to placebo ratio. The allocation sequence was generated using the pseudo-random number generator of the statistical software program SAS/STAT software (SAS Inc., version 9.13, Cary, NC) and used randomly varied permuted block sizes. Individual assignments were concealed in sequentially numbered, sealed, study drug kits. Randomization was stratified by site as determined on the date of enrollment. The participants, investigators, sponsor, and study staff did not know which gel a participant received, nor did they know the block size.

Enrolled participants received 14 identical, pre-filled, single-use applicators containing 3.5 g of one of the study products: VivaGel® or placebo gel. Fourteen additional applicators were given to participants at their Day 7 visit. Each applicator was over-wrapped in a sealed plastic envelope. The first application of study product was supervised in the clinic. The participant was instructed to insert the study product two times per day (one applicator in the morning and a second applicator in the evening) and to place the used applicator back into the overwrap and plastic envelope, and return them to the study clinic. The participant was also instructed to not use any other vaginal products and to abstain from sexual intercourse. She was given a diary card on which to record the date and time of product application, any symptoms experienced, and episodes of intercourse. In order to avoid having the gel interfere with the colposcopic assessments and laboratory testing, participants were instructed to not insert study gel at least 6 hours before a scheduled follow-up visit.

Participants returned to the clinic for follow-up visits on Day 2, Day 7, Day 14 and Day 21 following the enrollment visit. During follow-up visits, study staff collected used applicators, which were tested for exposure to vaginal mucus using trypan blue staining technique [15]. A review of symptoms, a pelvic exam including naked eye examination, and collection of vaginal samples for pH, wet mount, PSA (or Y-chromosome DNA), lactobacillus culture, and Gram stain were collected at each follow-up visit. Multiplex PCR testing for *Haemophilus Ducreyi*, *Treponema pallidum*, and HSV-2 was performed at each follow-up visit on participants with genital ulcers. Colposcopic examinations were conducted on the Day 2, Day 14, and Day 21 follow-up visits. In addition, participants with clinical GU signs or who reported GU symptoms on Day 7 received a colposcopic examination during that visit. During the Day 14 and Day 21 follow-up visit, urine was collected for pregnancy testing, and a blood sample was collected for hematology, liver and renal function testing, and to test for SPL7013 drug levels.

### Lab methods

Pregnancy was tested by rapid urine hCG assays (in Kisumu: Quidel QuickVue One-Step, Santa Clara, CA; in San Francisco: Mainline Confirms Pregnancy Rapid Assay, Mainline Technology, Inc, Ann Arbor, MI). Urine was tested by urine dipstick for evidence of urinary tract infection (Bayer Multistix, Deerfield, IL). HIV antibody testing was conducted using standardized algorithms at each site (in Kisumu: two different rapid tests were conducted (Abbott Determine HIV-1 and -2, Abbot Park, IL; and Unigold Rapid Assay, Trinity Biotech plc, Bray, Ireland), with a confirmatory ELISA (BioMerieux Vironostika HIV Uni-Form II Antigen/Antibody ELISA, Marcy l'Etoile, France); in San Francisco, an ELISA (BioMerieux Vironostika HIV Uni-Form II Antigen/Antibody ELISA) was conducted, with confirmatory Western Blot (Genetic Systems Reflex Western Blot by Bio-Rad, Hercules, CA) for ELISA positives). Syphilis was tested by rapid plasma reagin (Becton Dickenson Macrovue RPR, Franklin Lakes, NJ) with a confirmatory agglutination test (in Kisumu: Biokit Syphagen *T. pallidum* Hemaglutination, Barcelona, Spain; in San Francisco: Fujirebio Serodia *T. pallidum* Passive Particle Agglutination, Tokyo, Japan). HSV-2 antibody testing was conducted using an ELISA (Focus Technologies HSV-2 IgG ELISA, Cypress, CA). Bacterial vaginosis was determined through the evaluation of Gram stained vaginal smears using Nugent's criteria [13]. Vaginal swabs collected at enrollment and day 2 were evaluated for the presence of PSA on the ARCHITECT System (Abbott Laboratories, Abbott Park, Illinois) and vaginal samples with PSA concentrations ≥1 ng/mL were considered positive [14]. Vaginal swabs collected at days 7, 14, 21 were evaluated for sperm-derived Y chromosomal DNA sequences (Yc) using a Real-Time PCR assay (Roche Diagnostics, Indianapolis, Indiana) [16]. Cervicovaginal swabs were evaluated for *T. vaginalis*, *N. gonorrhoeae*, and *C. trachomatis* by Gen-Probe Aptima Combo 2 Assay (Gen-Probe Inc, San Diego, CA). Pap smears were conducted on cervical specimens (ThinPrep pap test, Cytyc Corporation, Marlborough, MA). Returned used applicators were tested for exposure to vaginal mucus using trypan blue staining technique [15]. Genital ulcers were swabbed and tested for H. *ducreyi*, *T. pallidum*, and HSV-2 by multiplex PCR. Vaginal cultures for semiquantitative microbiological assessment were obtained with polyester-tipped swabs and immediately placed in Port-A-Cul™ anaerobic transport tubes (Becton-Dickinson, Cockeysville, MD) and evaluated for the presence of lactobacillus and the production of hydrogen peroxide. Serum was tested for liver and kidney function and whole blood was used for complete blood counts and differentials. Pharmacokinetic analysis for plasma levels of SPL7013 was conducted by Starpharma using a capillary electrophoresis bioanalytical method employing a Micellar Electrokinetic Chromatography technique that had been developed and validated by Starpharma and which has a lower limit of quantitation for SPL7013 of 30 nM (0.5 µg/mL).

### Analysis of study outcomes

The planned sample size of 60 participants was based on recommendations stated in an update from the International Working Group on Microbicides [11]. Data entry, data management and statistical analysis were carried out by UCSF using SAS version 9.13 (SAS Institute Inc., Cary, NC). The analysis population was defined as participants who were randomized, and received at least one dose of study product, irrespective of whether they completed the study. Analyses for primary safety study endpoints were based on treatment group-specific summaries of frequency, severity and relationship to product use of AEs. AEs were tabulated both as frequencies and proportions of participants experiencing at least one event, and overall frequency of observed events pooled over participants. Within-participant changes in vaginal flora and laboratory parameters were also summarized for each treatment group. Formal comparisons between treatment groups in these outcomes were unadjusted and summarized using relative risks and associated 95% confidence intervals (CI).

## Results

### Enrollment and participant disposition

The planned sample size was 60 participants. However, on October 18, 2007 the study sponsor, the Division of Microbiology and Infectious Diseases (DMID), National Institute of Allergy and Infectious Diseases (NIAID), National Institutes of Health (NIH) made the decision to close the trial based on the belief that sufficient outcome data had been generated. This assessment was made following the pausing of a parallel study, Microbicides Trial Network (MTN)-004 sponsored by the Division of AIDS (DAIDS), NIAID, NIH, which had recently started to enrol participants in to a similarly designed trial of VivaGel® twice daily for 14 days at two sites, one in Tampa, Florida, and one in San Juan, Puerto Rico. After enrolling the first seven participants, the medical officer for MTN-004 decided to pause that trial so that an interim analysis of the data could be conducted, following several reports of genitourinary adverse events. The medical officer and project officer at DMID overseeing our trial made a decision based on this information, and the fact that we had enrolled 54 of the 60 intended participants, to close our trial. Of note, the MTN-004 trial protocol was subsequently amended, and the trial was restarted and completed [17]. Thus, a total of 54 women were enrolled in our trial, and among those, 5 women who were in active follow-up at the time the decision to close the trial was made were prematurely discontinued from study product and followed through their exit visit.

**Figure 1:**
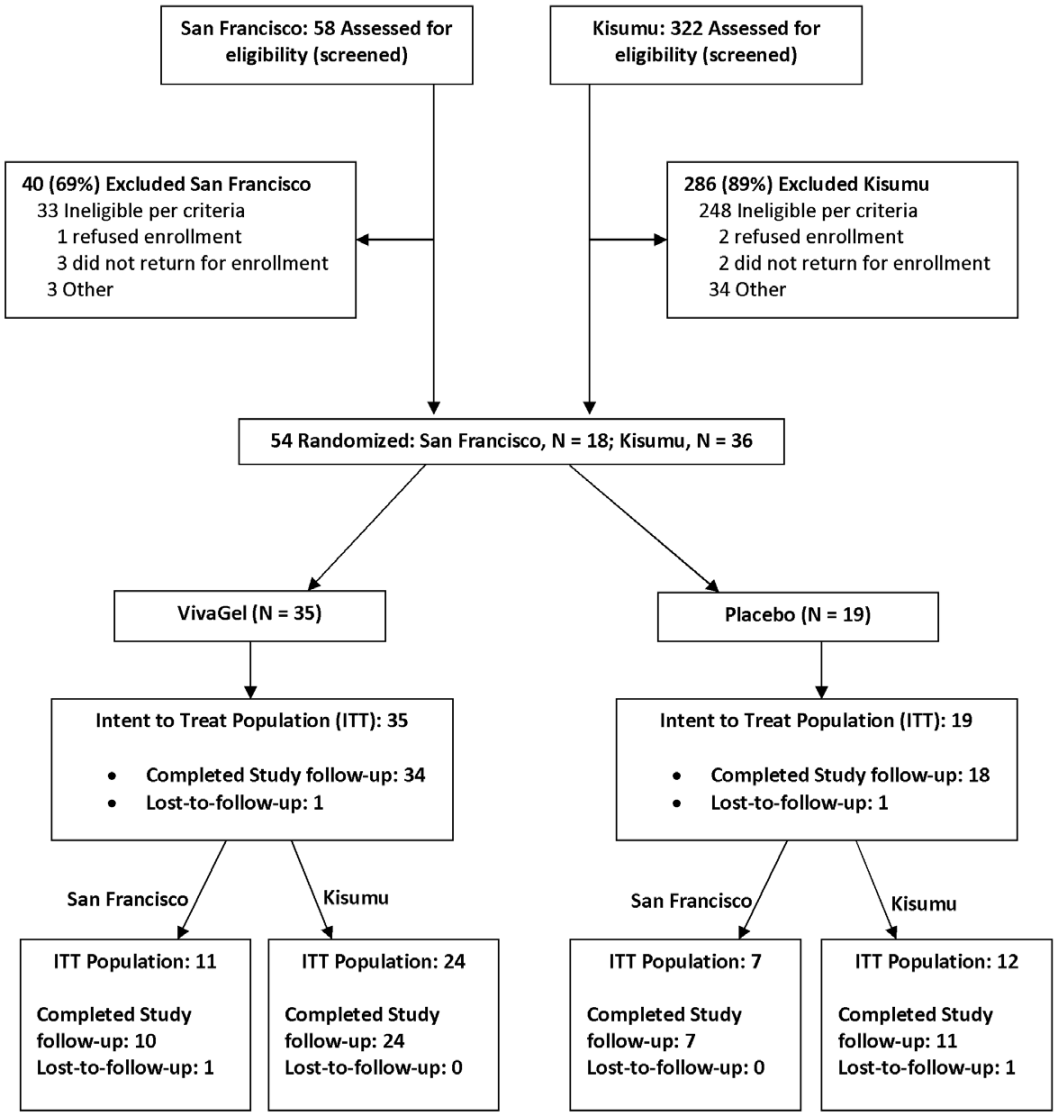
Flow of participants.

Of the 54 women, 35 women were enrolled in the VivaGel® arm and 19 in the placebo arm (Figure 1). One woman in each arm was lost to follow-up. Follow-up rates were high (89%–96%) at each visit, and were comparable between treatment arms. The mean age was 20.8 years in the VivaGel® arm vs. 20.9 years in the placebo arm (Table 1). Lifetime number of sexual partners was 2.6 (SD  = 2.7) and 4.1 (SD  = 5.9), and the number of sexual partners in the last 3 months was 0.8 (SD  = 0.5) and 0.9 (SD  = 0.7) in the VivaGel® arm and placebo arm, respectively (Table 1).

**Table 1:** Sociodemographic and sexual history by study arm and site.

	VivaGel® (SPL7013)			Placebo		
	SF	Kisumu	All	SF	Kisumu	All
	*N = 11 (%)*	*N = 24 (%)*	*N = 35 (%)*	*N = 7 (%)*	*N = 12 (%)*	*N = 19 (%)*
**Race/Ethnicity**						
American Indian/Alaska Native	0	0	0	0	0	0
Asian	3 (27%)	0	3 (8.5%)	0	0	0
Native Hawaiian/Other PacificIslanders	0	0	0	0	0	0
Black/African American	0	0	0	1 (14%)	0	1
White	8 (73%)	0	8 (23%)	5 (71%)	0	5
Other racialcategory	0	0	0	1 (14%)	0	1
African (Kenyan)	0	24 (100%)	24 (68.5%)	0	12 (100%)	12
Age (years)	21.6 (2.2)	20.5 (1.5)	20.8 (1.8)	21.7 (1.6)	20.4 (1.7)	20.9 (1.8)
Lifetime number of sexual partners (mean, SD)	4.7 (4.0)	1.6 (0.8)	2.6 (2.7)	8.1 (8.6)	1.8 (0.8)	4.1 (5.9)
Number of sexual partners last 3 mo (mean, SD)	0.9 (0.3)	0.8 (0.5)	0.8 (0.5)	1.1 (1.1)	0.8 (0.5)	0.9 (0.7)

### Adherence and tolerability

Adherence data included participants who discontinued study product for any reason, including those instructed to discontinue study product by study staff. Overall, adherence was higher in the VivaGel® arm compared to the placebo arm; twenty-six (74%) participants and 10 (53%) participants, respectively, reported taking all 28 doses of VivaGel® and placebo (Table 2). Adherence according to the protocol was similar between the VivaGel®(7 (64%)) and placebo (4 (57%)) arms at the San Francisco site, while at the Kisumu site adherence was greater among participants in the VivaGel® arm (19 (79%)) in comparison to the placebo arm (6 (50%)).

**Table 2:** Product adherence and tolerability by treatment group as per protocol definitions.

	VivaGel® (SPL7013)			Placebo		
	N	(%)	Number of doses taken	N	(%)	Number of doses taken
**All 28 doses taken as defined per protocol**	**26**	**(74.2)**		**10**	**(52.6)**	
**Premature product discontinuation by study staff**	**2**	**(5.7)**		**4**	**(21.0)**	
* Adverse Event*	0	--	--	0	--	--
* Closure of trial*	1	(2.9)	4	2	(10.5)	7,14
* Protocol deviation*	1*	(2.9)	10	2*	(10.5)	11,24
**Tolerability: Premature product discontinuation by study participant due to reported adverse event**	**2**	**(5.7)**		**0**	**--**	
* Gross hematuria*	1†	(2.9)	14	0	--	--
* Pelvic pain and cramping, leakage of gel*	1	(2.9)	22	0	--	--
**Imperfect adherence per protocol for other reason**	**4**	**(11.4)**		**4**	**(21.1)**	
* Participant forgot*	1	(2.9)	26	2	(10.5)	24, 27‡
* Participant started menses*	1	(2.9)	25	1	(5.3)	24
* Participant misplaced applicator*	1†	(2.9)	27	1	(5.3)	27
* Participant took 3 doses on one day (not per protocol)*	1	(2.9)	27	0	**--**	**--**
**Lost-to-follow-up**	**1**	**(2.9)**	**--**	**1**	**(5.3)**	13

*One enrolled participant was later found to not meet eligibility criteria and was subsequently discontinued from product use, one enrolled participant was erroneously thought to not meet eligibility criteria and was discontinued from product use, and one participant was discontinued from product use by the site PI after completing her Day 14 visit.

† These participants were subsequently discontinued from product due to closure of trial.

‡ This participant took all 28 doses in 16 days, but missed one or both doses on three days, and thus was not adherent per the protocol definition.

No participant was discontinued from study product by study staff due to an AE. Two (5.7%) participants in the VivaGel® arm prematurely discontinued themselves from study product due to concerns with symptoms; one reported discontinuing study product due to gross hematuria after taking 14 doses, and the other reported discontinuing study product due to pain during last product insertion, cramping and gel leakage after taking 22 doses (Table 2). In addition, 2 (5.7%) participants in the VivaGel® arm and 4 (21.0%) in the placebo arm did not take all 28 doses due to product discontinuation by study staff; 3 due to early closure of the study, and 3 due to protocol deviations by study staff (Table 2). Four (11.4%) participants in the VivaGel® arm and 4 (21.1%) in the placebo arm did not take all 28 doses per protocol due to other reasons (Table 2). At the San Francisco site a lower proportion of participants took one or more of the assigned VivaGel® doses for ≥8 days (82%) in comparison to the placebo (100%), while at the Kisumu site a greater proportion of participants in the VivaGel® arm took one or more of the assigned doses for ≥8 days (92%) in comparison to the placebo (67%). Dosing information is unavailable for 1 (2.9%) participant in the VivaGel® arm (San Francisco) and 1 (5.3%) in the placebo arm (Kisumu) who were lost to follow-up after enrollment.

Study participants returned 99% of used applicators to the study clinic. Of the returned used applicators, 97% of both the VivaGel® and placebo applicators stained positive for exposure to vaginal mucus, and staining results did not vary meaningfully by site (99% and 97% of used applicators stained positive in San Francisco and Kisumu, respectively).

Despite repeated counseling at each study visit to abstain from sexual intercourse for 7 days prior to enrollment and throughout the study period, one participant in the VivaGel® arm reported having sexual intercourse during the study period. In addition, 4 (11.4%) participants in the VivaGel® arm and 3 (15.7%) participants in the placebo arm tested positive for exposure to sperm by PSA or Y-chromosome testing which was performed after the conclusion of the trial; one participant at enrollment, two at Day 7, three at Day 14, and one at Day 21 (data not shown).

### Genitourinary Adverse Events

No grade 3 or 4 AEs, or SAEs were detected. Over the course of the trial, 106 grade 1 or 2 GU AEs (associated and not-associated with product use) were reported by 29 (83%) participants in the VivaGel® arm compared to 33 reported in 11 (58%) participants in the placebo arm. The relative risk (RR) comparing these proportions between the VivaGel® and placebo arms was 1.4 (95% CI 0.95-2.2) (Table 3). Limiting the analysis only to GU AEs associated with product use, 83 grade 1 or 2 GU AEs were reported by 25 (71%) participants in the VivaGel® arm compared to 20 reported in 10 (53%) participants in the placebo arm. The relative risk comparing these proportions between the VivaGel® and placebo arms was 1.4 (95% CI 0.8-2.2) (Table 3). Vulvar and cervical erythema, cervical lesions, clinically diagnosed symptomatic BV, urinary frequency, metrorrhagia, and unexplained infrequent bleeding were more common among participants in the VivaGel® arm than the placebo arm (see Table S1). Vaginal and vulval pain was reported more frequently in the placebo arm than the VivaGel® arm (see Table S1). During the second and third weeks of follow-up (i.e. Days 8–14 and Days 15–24), but not during the first week of follow-up (i.e. Days 0–7) the proportion of women with a GU AE associated with product use was greater among women in the VivaGel® arm than in the placebo arm (Table 3). Two participants in the VivaGel® arm had grade 1 perianal ulcers, both of which were negative for HSV-2, *H. ducreyi*, and *T. pallidum*. Although relatively rare, all grade 2 GU AEs associated with product use (n = 7) were experienced by 4 participants in the VivaGel® arm at the San Francisco site.

**Table 3:** The number of participants that experienced at least one, and the total number of genitourinary (GU) and laboratory adverse events* by treatment group, initiation day of AE, and association with study product.

	VivaGel® (SPL7013) (N = 35)						Placebo (N = 19)					
	assoc.		not assoc.		total		assoc.		not assoc.		total	
	% (*N*)	*TN*	% (*N*)	*TN*	% (*N*)	*TN*	% (*N*)	*TN*	% (*N*)	*TN*	% (*N*)	*TN*
**GU and Lab AEs, total**	83 (29)	94	57 (20)	27	91 (32)	121	74 (14)	28	58 (11)	16	79 (15)	44
GU AEs, total	71 (25)	83	51 (18)	23	83 (29)	106	53 (10)	20	47 (9)	13	58 (11)	33
* *During days 0–7	46 (16)	40	40 (14)	16	60 (21)	56	42 (8)	11	32 (6)	8	58 (11)	19
* *During days 8–14	37 (13)	29	6 (2)	2	40 (14)	31	21 (4)	5	11 (2)	2	32 (6)	7
* *During days 15–24†	29 (10)	14	9 (3)	5	34 (12)	19	16 (3)	4	11 (2)	3	26 (5)	7

N =  number of women with at least one finding; TN  =  total number of findings.

*Adverse events that did not occur are not listed.

† Includes 11 events on Day 22 and one each on Days 23 and 24.

At the San Francisco site a slightly greater proportion of participants in the placebo arm compared with the VivaGel® arm reported at least one GU AE associated with study product. A total of 52 GU AEs considered associated with study product were reported by 10 (91%) participants in the VivaGel® in comparison to 31 GU AEs reported by 7 (100%) in the placebo arm among participants at the San Francisco site. In contrast, among participants at the Kisumu site a greater proportion of participants had at least one GU AE associated with product use in the VivaGel® arm: 31 GU AEs were reported in 15 (63%) participants in comparison to the placebo arm where 6 GU AES were reported in 3 (25%) participants at the Kisumu site. It is of note that participants at the San Francisco site recruited half the number of participants than Kisumu, yet reported considerably higher numbers of GU AEs. Specifically, abnormal vaginal discharge (not including gel leakage) was reported more commonly among women enrolled in San Francisco (83%) than in Kisumu (19%), although the proportion of women reporting abnormal vaginal discharge (not including gel leakage) was similar among women in the VivaGel® and placebo arms in San Francisco (82% vs. 86%, respectively) and Kisumu (17% vs. 8%, respectively).

### Colposcopy findings

Over the course of the trial, 29 (83%) participants in the VivaGel® arm had 77 colposcopic findings compared to 10 (53%) participants having 20 such findings in the placebo arm (Table 4). The proportion of women who had a colposcopic finding was significantly greater among women in the VivaGel® arm than in the placebo arm (83% vs. 53%; RR = 1.6, 95% CI 1.0-2.5). Colposcopy findings were designated as either potentially related or unrelated to speculum insertion. In the VivaGel® arm 16 (46%) women had 26 colposcopic findings potentially related to speculum insertion compared to 5 (26%) participants with 7 potentially related findings in the placebo arm. In regards to colposcopy finding unrelated to speculum insertion 21 (60%) participants had 51 findings in the VivaGel® arm compared to 8 (42%) participants with 13 findings in the placebo arm. Overall, the proportion of women with at least one colposcopic finding was similar across study sites; 79% and 91% of women in the VivaGel® arm compared to 50% and 57% of women in the placebo arm in Kisumu and San Francisco, respectively had at least one colposcopic finding during follow-up. In this study, the majority (78/81 (97%)) of colposcopy findings were superficial. One woman in the VivaGel® arm had two deep epithelial lesions while one woman in the placebo arm had a single deep epithelial disruption during follow-up (Table 4).

**Table 4:**
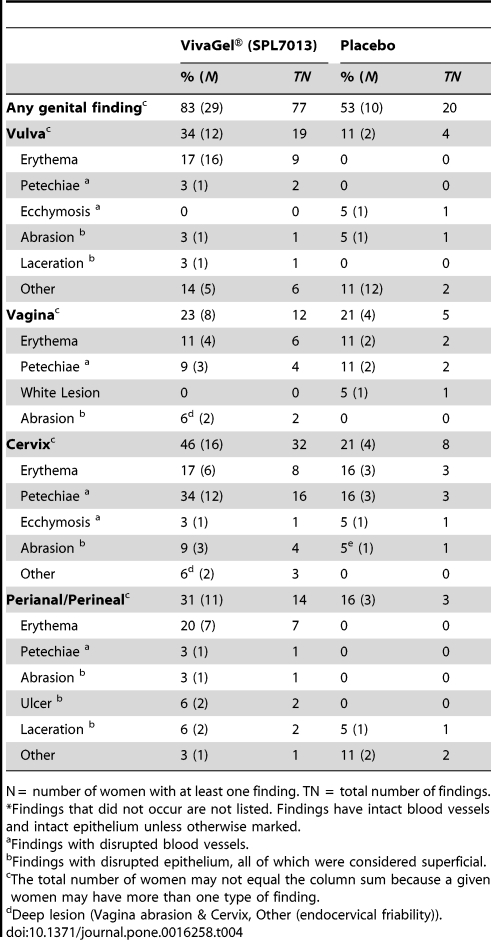
The number of participants that had at least one, and the total number of colposcopic findings both related and unrelated to speculum use* by treatment group.

N =  number of women with at least one finding. TN  =  total number of findings.

*Findings that did not occur are not listed. Findings have intact blood vessels and intact epithelium unless otherwise marked.

a Findings with disrupted blood vessels.

b Findings with disrupted epithelium, all of which were considered superficial.

c The total number of women may not equal the column sum because a given women may have more than one type of finding.

d Deep lesion (Vagina abrasion & Cervix, Other (endocervical friability)).

### Vaginal Microflora

Ninety seven percent and 95% of participants in the VivaGel® and placebo arms, respectively had *lactobacillus sp*. present at enrollment. Throughout the course of the trial 86% of participants in the VivaGel® arm and 68% of participants in the placebo arm had lactobacilli present at every visit, with the majority of women found with H_2_O_2_-non-producing lactobacilli at every visit (63% among women in the VivaGel® arm and 58% among women in the placebo arm). Persistence of H_2_O_2_-producing and H_2_O_2-_non-producing lactobacilli was common throughout the course of the trial, and did not differ by study arm. In general, lactobacillus status (presence/absence) was stable in women during the treatment period and post-dosing phase. Five (9%) participants had BV diagnosed by Nugent's criteria at enrollment (3 (9%) participants in the VivaGel® group, and 2 (11%) in the placebo group, all at the Kisumu site). In all cases, BV resolved after 7 days of antibiotic treatment according to local standard of care. The onset of symptomatic BV diagnosed by Amsel's criteria during follow-up was found among 4 (11%) participants who were all in the VivaGel® arm; two of these cases of BV were among participants from Kisumu who had enrolled in the study with asymptomatic BV by Nugent's criteria.

### Non-genitourinary adverse events and laboratory findings

Nervous system and gastrointestinal disorders were the most common non-GU AEs by organ system. Except for headache, which was more common in participants in the VivaGel® arm, non-GU AEs did not appear different between study groups. AEs due to changes in serum chemistry and liver and renal panels occurred infrequently and appeared fairly balanced between the two arms of the trial (Table 3). No participant had a positive pregnancy test. SPL7013 was not detected in the plasma of any participant.

## Discussion

This study assessed the safety and tolerability of VivaGel® versus placebo gel when used vaginally twice a day for 14 days in 18 to 24 year old women in San Francisco and Kisumu. GU AEs were common and occurred in both arms; 83% of participants in the VivaGel® arm and 58% of participants in the placebo arm experienced at least one grade 1 or 2 GU AE. Overall, GU AEs judged to be associated with product use were more common among participants in the VivaGel® arm than the placebo arm. These AEs were predominantly mild in nature (grade 1) and were self-limiting. All grade 2 GU AEs that were associated with product use occurred in four women in the VivaGel® arm at the San Francisco site. Superficial colposcopic abnormalities were also common in both arms and these findings were detected more commonly among participants in the VivaGel® arm. Vaginal flora was not adversely affected by study product and there were no significant changes to colonization with *lactobacillus spp*. Changes in serum chemistry and renal and liver function tests occurred infrequently during follow-up across both treatment arms and are unlikely to be related to study treatment since it was also found that there was no evidence of systemic absorption of SPL7013. There were no grade 3 or 4 AEs. Two participants in the VivaGel® arm discontinued themselves from study product during follow-up due to a self-reported GU AE. No participant was discontinued from study product by the study staff due to an AE.

The proportion of women with a GU AE associated with product use was greater among those randomized to the VivaGel® arm in comparison to the placebo arm among participants enrolled at the Kisumu site but not at the San Francisco site. The Kisumu site enrolled twice as many participants as the San Francisco site, and with a 2∶1 randomization scheme, the number of women assigned to the placebo arm was relatively small at each site. Adherence in the VivaGel® group was higher than in the placebo group at the Kisumu site, contributed in part by the early closure of the study and the discontinuation of study product in a greater proportion of those randomized to the placebo arm. Thus it is possible that exposure to fewer doses of placebo in comparison to VivaGel® among participants at the Kisumu site may have contributed to the difference in the incidence of GU AEs. Both VivaGel® and the placebo are carbopol-based aqueous gels. Thus, if carbopol or an excipient in the gel led to GU AEs, it is possible that the differences in GU AEs observed between the VivaGel® and placebo arms may in part be due to differential exposure to the investigational product between the study arms in Kisumu. Alternatively, young women in Kisumu might be more susceptible to epithelial irritation in the genital tract perhaps in relation to differences in vaginal flora or increased immune activation in their genital mucosa [18]. Furthermore, imperfect adherence to product per protocol in the VivaGel® (74%) and placebo (53%) arms may have resulted in an underestimation of the true risk of AEs associated with twice daily product use over 14 days.

Results of this trial are consistent with previously published phase 1 vaginal microbicide studies among sexually abstinent populations in that GU AEs were common and generally mild. In the phase 1 trial of once daily dosing for seven days of SPL7013, GU AEs considered potentially product-related were all mild and reported by 5 out of 25 (20%) women receiving VivaGel® (0.5%, 1.0% or 3.0%) and 2 (17%) of 12 women receiving placebo gel [10]. In published reports of other candidate vaginal microbicides the proportion of participants who experienced a GU AE has ranged from 14% to 87% [19]–[25]. To our knowledge, this is one of the first published studies to use the newly developed toxicity table to grade GU AEs in microbicide clinical trials which could theoretically have contributed to the high proportion of women found to experience GU AEs in this study. It is unlikely that the number of GU AEs reported was due to the young age of our participants and this is supported by a recently published study that found that older age was positively correlated with the proportion of participants with a genital finding in another microbicide trial [26]. The high rate of reported AEs and colposcopic findings in the placebo arm may suggest that the vehicle itself may have caused mild irritation. In addition, participants in San Francisco reported a greater incidence of AEs in comparison to participants in Kisumu. This finding could have arisen from a difference in perception of AEs, for example women in San Francisco reported more abnormal vaginal discharge than women in Kisumu, or may represent biological differences between these distinct populations. Regardless, this finding suggests the importance of conducting multisite trials in different populations to help evaluate product safety.

The strengths of this study include the double-blind placebo controlled design, and the inclusion of biomarkers to corroborate self-reported use of study product and sexual abstinence. The study population was limited to women aged 18–24 years as they are in greatest need of a microbicide to prevent HIV and STIs; however, these findings may not be generalizable to older age groups. By simultaneously conducting this study in the U.S. and Kenya we have collected safety data in populations of varying characteristics, an important step in the clinical development of any vaginal microbicide.

GU AEs and colposcopic findings consistent with mild genital epithelial irritation and inflammation occurred more commonly in the VivaGel® arm compared with placebo gel when the products were used twice daily for 14 days. Whether the findings from this study translate into safety concerns for this product cannot be ascertained entirely from this study, but should be assessed in conjunction with all the available preclinical and clinical data.

## Supporting Information

Table S1 Supplement to Table 3.
(DOC)
