# Supplementary material for: A Phase I Randomized Placebo Controlled Trial of the Safety of 3% SPL7013 Gel (VivaGel®) in Healthy Young Women Administered Twice Daily for 14 Days
Source: PLoS One. 2011 Jan 20;6(1):e16258. doi: 10.1371/journal.pone.0016258 (PMC3024437; doi:10.1371/journal.pone.0016258)
Supplement: Table S1 — Supplement to Table 3. (DOC) [file pone.0016258.s001.doc]

**Supplemental to Table 3. The number of participants that experienced at least one, and the total number of genitourinary (GU) and laboratory adverse e**vents* by treatment group and association with study product

|  | **VivaGel**® **(SPL7013) (N = 35)** | | | | | | | **Placebo (N = 19)** | | | | | | |
| --- | --- | --- | --- | --- | --- | --- | --- | --- | --- | --- | --- | --- | --- | --- |
|  | **assoc.** | | | **not assoc.** | | **total** | | **assoc.** | | | **not assoc.** | | **total** | |
|  | **% (*N*)** | ***TN*** | | **% (*N*)** | ***TN*** | **% (*N*)** | ***TN*** | **% (*N*)** | | ***TN*** | **% (*N*)** | ***TN*** | **% (*N*)** | ***TN*** |
| **GU and Lab AEs, total** | 83 (29) | 94 | | 57 (20) | 27 | 91 (32) | 121 | 74 (14) | | 28 | 58 (11) | 16 | 79 (15) | 44 |
| GU AEs, total | 71 (25) | 83 | | 51 (18) | 23 | 83 (29) | 106 | 53 (10) | | 20 | 47 (9) | 13 | 58 (11) | 33 |
| During days 0-7 | 46 (16) | 40 | | 40 (14) | 16 | 60 (21) | 56 | 42 (8) | | 11 | 32 (6) | 8 | 58 (11) | 19 |
| During days 8-14 | 37 (13) | 29 | | 6 (2) | 2 | 40 (14) | 31 | 21 (4) | | 5 | 11 (2) | 2 | 32 (6) | 7 |
| During days 15-24† | 29 (10) | 14 | | 9 (3) | 5 | 34 (12) | 19 | 16 (3) | | 4 | 11 (2) | 3 | 26 (5) | 7 |
| **Grade 1** (mild) |  | | | | | | | | | | | | | |
| Genital tract pain |  |  |  | |  |  |  |  |  | |  |  |  |  |
| -pelvic pain | 0 | 0 | 6 (2) | | 2 | 6 (2) | 2 | 0 | 0 | | 11 (2) | 2 | 11 (2) | 2 |
| -vaginal & vulvar pain | 6 (2) | 4 | 6 (2) | | 2 | 11 (4) | 6 | 11 (2) | 2 | | 11 (2) | 2 | 16 (3) | 4 |
| -dysmenorrhea | 0 | 0 | 0 | | 0 | 0 | 0 | 0 | 0 | | 5 (1) | 1 | 5 (1) |  |
| Vulva |  |  |  | |  |  |  |  |  | |  |  |  |  |
| -itching | 6 (2) | 2 | 0 | | 0 | 6 (2) | 2 | 5 (1) | 1 | | 0 | 0 | 5 (1) | 1 |
| -erythema | 17 (6) | 7 | 3 (1) | | 1 | 20 (7) | 8 | 5 (1) | 1 | | 0 | 0 | 5 (1) | 1 |
| -lesions | 11 (4) | 4 | 3 (1) | | 1 | 11 (4) | 5 | 11 (2) | 2 | | 5 (1) | 1 | 16 (3) | 3 |
| -abrasions | 3 (1) | 1 | 0 | | 0 | 3 (1) | 1 | 0 | 0 | | 0 | 0 | 0 | 0 |
| Vagina |  |  |  | |  |  |  |  |  | |  |  |  |  |
| -itching | 6 (2) | 3 | 0 | | 0 | 6 (2) | 3 | 0 | 0 | | 0 | 0 | 0 | 0 |
| -discharge | 37 (13) | 15 | 6 (2) | | 2 | 43 (15) | 17 | 37 (7) | 10 | | 5 (1) | 1 | 37 (7) | 11 |
| -lesions | 3 (1) | 1 | 0 | | 0 | 3 (1) | 1 | 0 | 0 | | 0 | 0 | 0 | 0 |
| Cervix |  |  |  | |  |  |  |  |  | |  |  |  |  |
| - edema | 3 (1) | 1 | 0 | | 0 | 3 (1) | 1 | 0 | 0 | | 0 | 0 | 0 | 0 |
| -erythema | 9 (3) | 3 | 9 (3) | | 3 | 17 (6) | 6 | 0 | 0 | | 0 | 0 | 0 | 0 |
| -lesions | 17 (6) | 7 | 6 (2) | | 2 | 20 (7) | 9 | 5 (1) | 1 | | 5 (1) | 1 | 11 (2) | 2 |
| Perianal |  |  |  | |  |  |  |  |  | |  |  |  |  |
| -erythema | 3 (1) | 1 | 0 | | 0 | 3 (1) | 1 | 5 (1) | 1 | | 0 | 0 | 5 (1) | 1 |
| -laceration | 0 | 0 | 3 (1) | | 1 | 3 (1) | 1 | 0 | 0 | | 0 | 0 | 0 | 0 |
| -irritation | 6 (2) | 2 | 3 (1) | | 1 | 9 (3) | 3 | 0 | 0 | | 0 | 0 | 0 | 0 |
| Urinary tract |  |  |  | |  |  |  |  |  | |  |  |  |  |
| -urinary frequency | 9 (3) | 5 | 3 (1) | | 1 | 11 (4) | 6 | 0 | 0 | | 0 | 0 | 0 | 0 |
| -dysuria | 3 (1) | 1 | 0 | | 0 | 3 (1) | 1 | 0 | 0 | | 5 (1) | 1 | 5 (1) | 1 |
| Vulvovaginitis | 3 (1) | 1 | 0 | | 0 | 3 (1) | 1 | 0 | 0 | | 0 | 0 | 0 | 0 |
| Cervicitis | 6 (2) | 2 | 0 | | 0 | 6 (2) | 2 | 0 | 0 | | 0 | 0 | 0 | 0 |
| Candida | 0 (0) | 0 | 0 (0) | | 0 | 0 (0) | 0 | 0 (0) | 0 | | 0 | 0 | 0 (0) | 0 |
| Bacterial vaginosis (clinical) | 11 (4) | 4 | 0 | | 0 | 11 (4) | 4 | 0 | 0 | | 0 | 0 | 0 | 0 |
| Metrorrhagia | 23 (8) | 8 | 17 (6) | | 6 | 40 (14) | 14 | 11 (2) | 2 | | 11 (2) | 2 | 21 (4) | 4 |
| Unexplained infrequent bleeding | 6 (2) | 3 | 3 (1) | | 1 | 9 (3) | 4 | 0 | 0 | | 0 | 0 | 0 | 0 |
| Post-coital bleeding | 3 (1) | 1 | 0 | | 0 | 3 (1) | 1 | 0 | 0 | | 0 | 0 | 0 | 0 |
| Ruptured cyst | 0 | 0 | 0 | | 0 | 0 | 0 | 0 | 0 | | 5 (1) | 1 | 5 (1) | 1 |

| **Table 3. continued** | | | | | | | | | | | | |
| --- | --- | --- | --- | --- | --- | --- | --- | --- | --- | --- | --- | --- |
|  | **VivaGel**® **(SPL7013)** | | | | | | **Placebo** | | | | | |
|  | **assoc.** | | **Not assoc.** | | **total** | | **assoc.** | | **not assoc.** | | **total** | |
|  | **% (*N*)** | ***TN*** | **% (*N*)** | ***TN*** | **% (*N*)** | ***TN*** | **% (*N*)** | ***TN*** | **% (*N*)** | ***TN*** | **% (*N*)** | ***TN*** |
| Protocol specified laboratory abnormalities |  |  |  |  |  |  |  |  |  |  |  |  |
| -hemoglobin | 9 (3) | 3 | 3 (1) | 1 | 11 (4) | 4 | 0 | 0 | 5 (1) | 1 | 5 (1) | 1 |
| -WBC | 0 | 0 | 3 (1) | 1 | 3 (1) | 1 | 5 (1) | 1 | 5 (1) | 1 | 11 (2) | 2 |
| -monocytes | 0 | 0 | 3 (1) | 1 | 3 (1) | 1 | 0 | 0 | 0 | 0 | 0 | 0 |
| -creatinine | 6 (2) | 2 | 0 | 0 | 6 (2) | 2 | 0 | 0 | 0 | 0 | 0 | 0 |
| -bilirubin | 3 (1) | 1 | 0 | 0 | 3 (1) | 1 | 5 (1) | 1 | 0 | 0 | 5 (1) | 1 |
| -AST | 6 (2) | 2 | 0 | 0 | 6 (2) | 2 | 11 (2) | 2 | 0 | 0 | 11 (2) | 2 |
| -ALT | 3 (1) | 1 | 0 | 0 | 3 (1) | 1 | 0 | 0 | 0 | 0 | 0 | 0 |
| -ALKP | 3 (1) | 1 | 3 (1) | 1 | 6 (2) | 2 | 16 (3) | 3 | 0 | 0 | 16 (3) | 3 |
| **Grade 2** (moderate) |  | | | | | | | | | | | |
| Genital tract pain |  |  |  |  |  |  |  |  |  |  |  |  |
| -vaginal pain | 3 (1) | 1 | 0 | 0 | 3 (1) | 1 | 0 | 0 | 0 | 0 | 0 | 0 |
| Dyspareunia | 3 (1) | 1 | 0 | 0 | 3 (1) | 1 | 0 | 0 | 0 | 0 | 0 | 0 |
| Vagina – itching | 3 (1) | 1 | 0 | 0 | 3 (1) | 1 | 0 | 0 | 0 | 0 | 0 | 0 |
| Cervix –erythema | 3 (1) | 1 | 0 | 0 | 3 (1) | 1 | 0 | 0 | 0 | 0 | 0 | 0 |
| Cervicitis | 6 (2) | 2 | 0 | 0 | 6 (2) | 2 | 0 | 0 | 0 | 0 | 0 | 0 |
| Urinary Tract – hematuria | 3 (1) | 1 | 0 | 0 | 3 (1) | 1 | 0 | 0 | 5 (1) | 1 | 5 (1) | 1 |
| Laboratory abnormalities |  |  |  |  |  |  |  |  |  |  |  |  |
| -platelets | 3 (1) | 1 | 0 | 0 | 3 (1) | 1 | 0 | 0 | 0 | 0 | 0 | 0 |
| -hemoglobin | 0 | 0 | 0 | 0 | 0 | 0 | 5 (1) | 1 | 5 (1) | 1 | 11 (2) | 2 |

N = number of women with at least one finding; TN = total number of findings

MCV = mean corpuscular volume; WBC = white blood cells; AST = Aspartate transaminase; ALT = Alanine transaminase; ALKP = Alkaline phosphatase

*Adverse events that did not occur are not listed

†Includes 11 events on Day 22 and one each on Days 23 and 24.
